# Isolation, characterization, and molecular regulation of muscle stem cells

**DOI:** 10.3389/fphys.2013.00317

**Published:** 2013-11-12

**Authors:** So-ichiro Fukada, Yuran Ma, Takuji Ohtani, Yoko Watanabe, Satoshi Murakami, Masahiko Yamaguchi

**Affiliations:** Laboratory of Molecular and Cellular Physiology, Graduate School of Pharmaceutical Sciences, Osaka UniversityOsaka, Japan

**Keywords:** satellite cells, skeletal muscle, maintenance, muscular diseases, quiescence, notch, microarray, cell therapies

## Abstract

Skeletal muscle has great regenerative capacity which is dependent on muscle stem cells, also known as satellite cells. A loss of satellite cells and/or their function impairs skeletal muscle regeneration and leads to a loss of skeletal muscle power; therefore, the molecular mechanisms for maintaining satellite cells in a quiescent and undifferentiated state are of great interest in skeletal muscle biology. Many studies have demonstrated proteins expressed by satellite cells, including Pax7, M-cadherin, Cxcr4, syndecan3/4, and c-met. To further characterize satellite cells, we established a method to directly isolate satellite cells using a monoclonal antibody, SM/C-2.6. Using SM/C-2.6 and microarrays, we measured the genes expressed in quiescent satellite cells and demonstrated that Hesr3 may complement Hesr1 in generating quiescent satellite cells. Although Hesr1- or Hesr3-single knockout mice show a normal skeletal muscle phenotype, including satellite cells, Hesr1/Hesr3-double knockout mice show a gradual decrease in the number of satellite cells and increase in regenerative defects dependent on satellite cell numbers. We also observed that a mouse's genetic background affects the regenerative capacity of its skeletal muscle and have established a line of DBA/2-background *mdx* mice that has a much more severe phenotype than the frequently used C57BL/10-*mdx* mice. The phenotype of DBA/2-*mdx* mice also seems to depend on the function of satellite cells. In this review, we summarize the methodology of direct isolation, characterization, and molecular regulation of satellite cells based on our results. The relationship between the regenerative capacity of satellite cells and progression of muscular disorders is also summarized. In the last part, we discuss application of the accumulating scientific information on satellite cells to treatment of patients with muscular disorders.

## Introduction

One of the best-known examples of regeneration is the ability of newts to regenerate limbs and tails. This process was believed to utilize specialized multipotent cells that have the potential to produce all types of cells. The existence of this type of multipotent stem cells seemed to be the reason newts, but not humans, can regenerate limbs. However, a recent study of the axotl has suggested a different model (Kragl et al., 2009). Instead of multipotent stem cells, each tissue produces progenitor cells with restricted potential. In other words, each cell keeps a memory of its tissue origin during regeneration. Humans who lose a limb cannot produce new one because most types of cells do not retain their regenerative potentials in humans. Skeletal muscle, however, seems to be an exception. Muscle stem cells, known as satellite cells, make it possible for humans and newts to regenerate, because the potential for muscle regeneration using satellite cell-like cells is maintained across species (Morrison et al., 2006). Because the ultimate goal of regenerative medicine is to rebuild lost tissue, the study of muscle regeneration and satellite cells will help us clarify the principles of regeneration.

How many times can skeletal muscle rebuild itself? In other words, how many times can satellite cells proliferate to make new myofibers in mammals? Two groups have reported a powerful regenerative capacity of skeletal muscle in rats and mice. Luz et al. reported that C57BL/10 mice regenerated muscle without loss of myofibers or gain of fibrotic areas after 50 bupivacaine injections into the TA muscle (Luz et al., 2002). Sadeh et al. showed active regeneration cycles in rats that had received weekly injections of bupivacaine for 6 months. They reported a lack of evidence for reduction or exhaustion of muscle fiber capacity to regenerate despite ongoing degeneration–regeneration cycles over a period approximating one fourth of the rat life expectancy (Sadeh et al., 1985). These results clearly indicate that the satellite cell pool is efficiently maintained during multiple degeneration–regeneration cycles in these animals. However, actually, there are many inherited and non-inherited muscle disorders that exhibit a progressive loss of muscle mass and weakness. Duchenne muscular dystrophy is a well-known inherited muscle disease that results from the lack of functional dystrophin proteins. In disease environments, satellite cells are forced to continue to proliferate and differentiate because newly formed myofibers are repeatedly damaged. This regeneration–degeneration cycle is considered to lead to exhaustion of satellite cell potentials, which is one reason why dystrophic patients exhibit progressive symptoms. In addition, the microenvironment of satellite cells in disease conditions may affect long-term survival and/or maintenance of their functions. The same genes that are responsible for inherited muscle disease might directly contribute to sustaining the satellite cell pool. Our knowledge of how to overcome and develop new therapeutic methodologies for muscle diseases based on satellite cell biology is still limited, but if we can manipulate satellite cell potential, it may lead to treatment of many muscle disorders. To accomplish this, we have to understand the molecular and cellular mechanisms of satellite cells. In this chapter, we will introduce the methodology of direct isolation of satellite cells, which was the first step to revealing the molecular and cellular mechanisms of satellite cells. Next, based on recent studies, we will show molecular regulation of satellite cells, and the relationship between the capacity of satellite cells and the progression of muscular disorders. In the last part, we discuss how to apply the accumulating scientific results on satellite cells to treating patients with muscle disorders.

## Satellite cells

**Figure 1:**
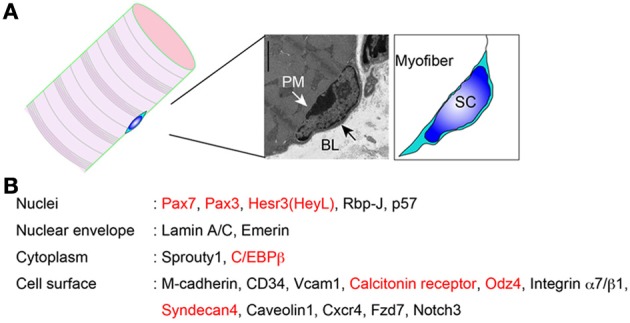
**Proteins expressed in quiescent muscle satellite cells. (A)** Location of satellite cells. PM, plasma membrane; BL, basal lamina; SC, satellite cell. **(B)** Satellite cell molecules whose protein expressions are confirmed. Red indicates “quiescence genes.”

Satellite cells were discovered by Dr. Alexander Mauro as mononuclear cells attached to myofibers in frog muscle (Mauro, 1961). Subsequently, satellite cells were found in mammalian skeletal muscle. The name is derived from their location between the basal lamina and sarcolemma (plasma membrane of myofiber) (Figure 1A). Like other stem cells, satellite cells are maintained in an undifferentiated and quiescent state in uninjured muscles (Schultz et al., 1978), and therefore, transcriptional activity is much lower than in proliferating myoblasts. In fact, the nucleus occupies most of the cell area, and only small portions are observed as cytoplasm by electronic microscopy (Figure 1A). In addition, the RNA content of quiescent satellite cells is about one fourth of that of cultured myoblasts (Fukada et al., 2007). Freter et al. demonstrated global suppression of RNA polymerase II serine-2 phosphorylation, which triggers productive transcription elongation, mRNA processing, and release of the mature mRNA in adult stem cells including satellite cells (Freter et al., 2010). However, recent studies show that the quiescent state is not “passive,” but rather a highly regulated cell state that is rapidly activated in response to injury or damage (Liu et al., 2013), therefore the “active” molecular regulation of stem cells is of great interest in the field of stem cell research.

In the past 15 years, many studies have reported other myogenic stem/progenitor cells, for example, bone marrow cells, side population (SP) cells, and muscle-resident interstitial cells, that seemed to function as stem cells for muscle regeneration (Ferrari et al., 1998; Gussoni et al., 1999; Jackson et al., 1999; Asakura and Rudnicki, 2002; Asakura et al., 2002; Fukada et al., 2002; LaBarge and Blau, 2002; Tamaki et al., 2002; Polesskaya et al., 2003). However, the physiological roles of these types of cells are limited, and, now there is no doubt that satellite cells play essential roles during skeletal muscle regeneration (Collins et al., 2005; Lepper et al., 2011; Sambasivan et al., 2011). In addition, a single satellite cell has potential for both myogenic differentiation and self-renewal; therefore, the functions and characteristics of satellite cells satisfy the criteria of stem cells, and satellite cells function physiologically as stem cells for skeletal muscle homeostasis (Sacco et al., 2008). Satellite cells also play essential roles in skeletal muscle development. White et al. estimated that satellite cells contribute to the increase in the number of myofiber nuclei in mice until 3 weeks after birth (White et al., 2010).

The identification and observation of satellite cells had been dependent on electronic microscopy. However, the discovery of M-cadherin expression on satellite cells has allowed us to easily identify them by conventional microscopy (Irintchev et al., 1994). Pax7 is also widely used to identify satellite cells by microscopy. In contrast to M-cadherin-null mice, Pax7-null mice show severe loss of muscle mass and satellite cell pools (Seale et al., 2000). In addition to these molecules, expression of several other proteins in quiescent satellite cells has been reported (Figure 1B). Above all, identification of cell surface molecules allows us to isolate living quiescent satellite cells, using a specific antibody that recognizes the extracellular domain of the protein. In the next section, we will introduce the history and methodologies of direct isolation of satellite cells.

## Direct isolation of satellite cells

Direct isolation of stem cells is a powerful tool to investigate their biology. Direct isolation of hematopoietic stem cells made research on hematopoietic stem cells the cutting edge of stem cell biology (Osawa et al., 1996). On the other hand, identification of stem cells by culture-based methods sometimes lacks reproducibility because culture conditions might affect it. Therefore, direct isolation of stem cells is essential for the study of stem cell biology.

When we started to study muscle satellite cells, nobody had succeeded in direct isolation of them, although Blau's group reported isolation of myogenic cells using an anti-integrin a7 antibody from crude cultured mononuclear cells derived from skeletal muscle (Blanco-Bose et al., 2001). To directly isolate satellite cells, we tried to develop a monoclonal antibody. We injected C2/4 cells (a subclone of C2C12) into rats, and produced hybridoma cells by a standard method. C2/4 cells were used for FACS-based screenings because our aim was to establish a monoclonal antibody suited to FACS analyses. In addition, skeletal muscle-derived mononuclear cells were also used for the FACS-based screening process. Finally, SM/C-2.6, a new monoclonal antibody, was established (Fukada et al., 2004), and then we established a method to isolate pure satellite cells using SM/C-2.6, anti-CD31, -CD45, and -Sca-1 antibodies (Fukada et al., 2007; Ikemoto et al., 2007). All mononuclear myogenic cells in skeletal muscle at different stages of injury as well as uninjured muscle were purified in the SM/C-2.6(+)CD31(−)CD45(−)Sca-1(−) fraction (Ikemoto et al., 2007; Segawa et al., 2008). SM/C-2.6 also contributed to the generation of myogenic cells from embryonic stem (ES) cells and induced pluripotent stem (iPS) cells (Chang et al., 2009; Mizuno et al., 2010). Furthermore, the antibody led to the identification of mesenchymal progenitors, which are the original cell sources of both the fibrosis and adipogenesis that are the hallmarks of progressed muscular dystrophies (Uezumi et al., 2010, 2011; Ito et al., 2013). In *in vitro* culture conditions, cells easily lose *in vivo* characteristics, including gene expression. In addition, a lack of purification allows contamination by other types of cells and affects the results. Therefore, the purification of isolated cells is essential for these analyses, and our methods to assure the purity are widely used in many laboratories (Israeli et al., 2007; Verma et al., 2010; Yajima et al., 2010; Tokura et al., 2011; Urciuolo et al., 2013).

There are other methods to purify muscle satellite cells. In 2004, Sherwood et al. demonstrated that the integrin α7(+)integrin β1(+)Cxcr4(+)CD34(+)CD45(−)Sca-1(−)Mac-1(−) fraction contained only myogenic cells (Sherwood et al., 2004). In 2005, Montarras et al. showed that satellite cells are highly enriched in the CD34(+)CD45(−)Sca-1(−) fraction (Montarras et al., 2005). Syndecan3/4 is also used as a positive marker of satellite cell isolation (Tanaka et al., 2009). The positive marker used depends on the laboratory, but many research groups use the same negative markers. The positive and negative markers used for directly isolating satellite cells are listed in Table 1. In addition to these cell surface-based methods for isolation of satellite cells, genetic modifications also allow us to directly isolate quiescent satellite cells. Green or yellow fluorescent protein expression under a Pax3 (Montarras et al., 2005; Bosnakovski et al., 2008) or Pax7 promoter is one established method for direct isolation of satellite cells. Unfortunately, although information on satellite cells in humans is still limited, SM/C-2.6 does not react with human, rat, or dog cells (unpublished data). Thus, neural cell adhesion molecules (NCAM) is used for identification of human satellite cells in tissues (Cashman et al., 1987), and a few groups have reported direct isolation of satellite cells using anti-NCAM (CD56) antibodies (Dellavalle et al., 2007).

**Table 1:** **Markers used in the direct isolation of muscle satellite cells**.

**Positive markers**	**Negative markers**	**References**
SM/C-2.6	CD31, CD45, Sca-1	Fukada et al., 2004, 2007
Integrin α7, Integrin β1, Cxcr4	CD45, Sca-1, Mac-1	Sherwood et al., 2004
CD34	CD45, Sca-1	Montarras et al., 2005

FACS analyses do not provide information about the location of satellite cells. In general, immunohistochemistry studies are necessary to provide information on the location of the cells of interest (Irintchev et al., 1994), and therefore, the expression of positive markers on satellite cells must be examined by immunohistochemistry in order to isolate satellite cells. Importantly, the positive markers for isolating satellite cells described above were examined their expression on satellite cells by immunohistochemistry. Therefore, isolated cells by FACS are considered to be equivalent to the anatomically identified satellite cells (Beauchamp et al., 2000; Cornelison et al., 2001; Fukada et al., 2004).

As described above, some muscle stem cells except for satellite cells are identified by FACS. Muscle-SP cells are defined by Hoechst-efflux (Gussoni et al., 1999; Jackson et al., 1999). Satellite cells do not exist in the SP cell fraction (Fukada et al., 2004), and therefore muscle satellite cells and SP cells are considered to be different cell populations. Like muscle-SP cells, the other types of muscle stem cells are located in interstitial areas in muscle (Tamaki et al., 2002; Uezumi et al., 2006), and therefore SP cells and muscle-resident interstitial cells are completely distinct populations from satellite cells, making the study of immunohistochemistry essential for using positive markers to isolate muscle satellite cells.

However, FACS studies have many advantages in on-going studies of stem cell biology. FACS can easily elucidate the cell size, cellular granularity, and frequency of a stem cell population (Fukada et al., 2011). In addition, direct isolation assures single cell transplantation (Sacco et al., 2008), and Montarras and Ikemoto reported that freshly isolated cells have much higher muscle reconstitution potential than cultured cells (Montarras et al., 2005; Ikemoto et al., 2007). Among the benefits of direct isolation, one of the most notable is that we can perform genome-wide gene expression analyses using isolated cells and microarrays. In fact, we have identified a great number of unexpected genes in quiescent satellite cells (Fukada et al., 2007). In the next section, we will introduce genes that are specifically or highly expressed in satellite cells in the dormant state.

## Quiescence genes

Cultured myoblasts and myogenic cell lines play important roles in studies of myogenic differentiation, and essential processes for myogenic differentiation have been established. Establishment of C2 cells (Yaffe and Saxel, 1977) and the subclone C2C12 (Blau et al., 1983a) has played extremely important roles in studies of myogenic cell biology. Single myofiber culture is also excellent model to investigate the differentiation and self-renewal mechanisms of satellite cells *in vitro* (Rosenblatt et al., 1995). However, little was known about the genes expressed in quiescent satellite cells until 2007 when our group first compared the genes of quiescent satellite cells and cultured myoblasts. We found that 507 genes (665 probes) were expressed in quiescent satellite cells at levels more than 5-fold higher than in activated satellite cells (Fukada et al., 2007). To date, among these genes, the physiological roles of Sprouty1 (Spry1), Notch3, and Cepbb, in satellite cells have been elucidated using gene-deleted mice (Kitamoto and Hanaoka, 2010; Shea et al., 2010; Marchildon et al., 2012). Spry1 is an inhibitor of receptor tyrosine kinase signaling, and the lack of Spry1 leads to loss of the satellite cell pool during the regeneration process. In uninjured young and adult muscle, Spry1 is not essential for maintaining satellite cells, but in aged muscle, the loss of Spry1 leads to a decrease in the number of satellite cells due to accelerated fibroblast growth factor (FGF) signaling (Chakkalakal et al., 2012). Kitamoto et al. showed that half of satellite cells and myoblasts express Notch3, and that Notch3 expression is downregulated during myogenic differentiation (Kitamoto and Hanaoka, 2010). Intriguingly, the loss of Notch3 increases the number of satellite cells in uninjured muscle. In addition, after repetitive muscle injuries, like repeated CTX injections or dystrophic condition, Notch3-deficient mice showed remarkable overgrowth of muscle mass. Notch3-deficient myoblasts show accelerated proliferation, and Notch3-induced myoblasts exhibit decreased BrdU-uptake. Therefore, in quiescent satellite cells, Notch3 might keep the cell cycle in a quiescent state. The investigation of aged Notch3-deficient mice is expected to explain the roles of Notch3 in satellite cells over long periods. CCAAT/enhancer binding proteins (C/EBPs) form a family of basic leucine zipper (bZIP) transcription factors, of which C/EBPβ is involved in many regulatory and differentiation processes as both an activator and a repressor. The loss of *Cebpb* does not affect the number of satellite cells, but C/EBPβ has the potential to induce Pax7 expression and inhibit myogenic differentiation because loss of *Cebpb* expression in satellite cells promotes fiber hypertrophy *in vivo* and cell fusion *in vitro* (Marchildon et al., 2012). Like Spry1, loss of *Cebpb* might affect the satellite cell pool in aged mice. In addition, our transcriptome analyses have shown that *Cebpd* is also highly expressed in quiescent satellite cells. Therefore, C/EBP family genes might regulate muscle satellite cells in a physiological manner.

Bone morphogenetic proteins (BMPs) constitute a subgroup of the transforming growth factor (TGF)-β superfamily, and are known as myogenic differentiation regulators. Gamell et al. reported that Bmp2 induces phosphorylation of Akt and migration of C2C12 (Gamell et al., 2008). Wang et al. indicated that Bmp signaling positively regulates the proliferation of both fetal myogenic progenitors and satellite cells *in vivo* (Wang et al., 2010). They also indicated that BMP signaling is not active in quiescent satellite cells, but that proliferating satellite cells exhibit active BMP signaling. These results indicate that quiescent satellite cells do not respond to BMP signaling in their dormant state. Intriguingly, our microarray results showed that quiescent satellite cells express extremely high levels of *Bmp2, 4*, and *6* genes (Fukada et al., 2007). Therefore, an unknown mechanism might control BMP activity or translation of BMPs in quiescent satellite cells. In BMP signaling, Smad1, Smad5, and Smad8 are specific intracellular transducers. On the other hand, Smad2 and Smad3 transduce TGF-β signaling. Ge et al. reported that Smad3-null mice showed a decreased number of satellite cells (Ge et al., 2011). However, this study was performed using Smad3-null mice, therefore, we cannot conclude that Smad3 plays direct roles in quiescent satellite cells because myofibers of Smad3-null mice are also affected by the lack of Smad3. However, hematopoietic stem cells (Yamazaki et al., 2011) and some stem cells (Oshimori and Fuchs, 2012) are controlled by TGF-β signaling. Likewise, TGF-β might be an essential regulator for the maintenance of satellite cells. In Table 2, we summarized the genes that affect satellite cell numbers in uninjured adult skeletal muscle (Seale et al., 2000; Kitamoto and Hanaoka, 2010; Angione et al., 2011; Fukada et al., 2011; Ge et al., 2011; Hosoyama et al., 2011; Juan et al., 2011; Bjornson et al., 2012; Chakkalakal et al., 2012; Cheung et al., 2012; Mourikis et al., 2012).

**Table 2:** **Genes known to control satellite cell number *in vivo***.

**Target molecule**	**Experimental procedure**	Result^*^	**References**
Pax7	Null	Decreased (60 days, 0–10%)	Seale et al., 2000
Hesr1 and Hesr3	Null	Decreased (20 weeks, <20%)	Fukada et al., 2011
Rbp-J	Conditional (Pax7-CreERT2)	Decreased (44 days, 20%)	Bjornson et al., 2012; Mourikis et al., 2012
Rb	Conditional (Pax7-CreERT2)	Increased (14 days, 152%)	Hosoyama et al., 2011
Notch3	Null	Increased (4 months, 140%)	Kitamoto and Hanaoka, 2010
Dicer	Conditional (Pax7-CreERT2)	Decreased (14 days, 20%)	Cheung et al., 2012
Smad3	Null	Decreased (50%)	Ge et al., 2011
Sprouty	Conditional (Pax7-CreERT2)	Decreased (22 months, 50%)	Chakkalakal et al., 2012
PPARd	Conditional (Myf5-Cre)	Decreased (2–3 months, 60%)	Angione et al., 2011
Ezh2	Conditional (Pax7-Cre)	Decreased (60 days, 60%)	Juan et al., 2011

* Percentage shows the frequency of satellite cell number compared to control mice. In the case of null mice, the period shows age of mice analyzed. In the case of Pax7-CreERT2 mice, the period after last injection of tamoxifen is indicated.

On the other hand, 659 genes (814 probes) were upregulated (>5-fold) in the activated state in our microarray results. The most highly upregulated gene (334-fold) was *Hmga2*; Li at al. demonstrated that Hmga2 plays essential roles in myoblast proliferation and myogenesis (Li et al., 2012). Therefore, our transcriptome analyses include many functional genes that explain satellite cell states.

As mentioned above, satellite cells occupy a unique location and do not express the myogenic determination gene MyoD. Therefore, we hypothesized that the genes responsible for maintaining satellite cells are specifically expressed in quiescent satellite cells in skeletal muscle. To isolate such genes, we also prepared non-myogenic cells from skeletal muscle for comparison with quiescent and activated satellite cells, and 63 genes were finally identified as “quiescence genes,” which are highly expressed in quiescent satellite cells but not in cultured myoblasts and non-myogenic cells in skeletal muscle (Fukada et al., 2007). Almost none of the genes had been previously reported in skeletal muscle biology.

Other groups also performed similar comparisons to characterize quiescent satellite cells. Pallafacchina et al. compared the gene expression profiles of quiescent satellite cells with samples of neonatal satellite cells and *mdx* mouse-derived Pax3+ cells (Pallafacchina et al., 2010). Our “quiescence genes” are expressed at higher rates in quiescent satellite cells than in neonatal satellite cells, but the ratio is not very significant. This discrepancy is dependent on the type of cells used for comparison because Pallafacchina et al. also showed significant differences between quiescent satellite cells and cultured myoblasts. Pallafacchina et al. isolated quiescent satellite cell-specific genes (e.g., *Apoe, Ms4a4d, Fgl2, Timp4, Adh1, Ahr, Osmr*), but these genes are also expressed in non-myogenic cells in skeletal muscle. Another discrepancy is the expression of Notch-related genes in quiescent satellite cells. We identified Notch signaling-related genes (*Notch3* and *HeyL/Hesr3*) as the most highly expressed genes in quiescent satellite cells, although Pallafacchina's results did not show the importance of Notch signaling in quiescent satellite cells. However, recent studies have clearly demonstrated the essential roles of Notch signaling for maintaining quiescent satellite cells *in vivo* as well as developmental stages (see below). On the other hand, quiescent satellite cells share common features in our and Pallafacchina's results, for example, some cell adhesion molecules and transcriptional factors. Although our reports had not mentioned it, Pallafacchina et al. intriguingly found up-regulation of anti-oxidative genes in quiescent satellite cells. In fact, our original data also included ant-oxidative genes in quiescent-stage specific manner. These results imply the importance of oxidative stress in quiescent satellite cells. Farina et al. analyzed gene expressions of quiescent satellite cells, activated satellite cells (ASC, 12 h after injury), and proliferating myoblasts (Prof. SC, 48 h after injury) (Farina et al., 2012). Their study focused mainly on the RNA-binding proteins that are highly expressed in quiescent satellite cells compared to ASC or Prof. SC. However, *Zfp36* was the sole common RNA-binding protein among the three microarray studies. Transcriptional and translational regulation of RNA may be essential for maintaining satellite cells in a dormant state, so quiescent satellite cell-specific expressions of RNA-binding proteins were limited in the three studies. The three microarray studies are summarized in Table 3. In the next sections, we would like to introduce our three “quiescence genes”: Hesr3, calcitonin receptor, and Odz4, with some speculation about their roles.

**Table 3:** **Molecular signatures of quiescent satellite cells identified by three independent studies**.

	**Fukada et al. (2007)**	**Pallafacchina et al. (2010)**	**Farina et al. (2012)**
**Comparison**	Cultured myoblasts, NON-myogenic cells	1-week neonatal SC, *mdx* SC	*In vivo* activated SC12, 48 h, Syndecan-4-null
**Method**			
Isolation	SM/C-2.6+CD45-	Pax3-GFP	Syndecan-3
Muscle	Limb muscles	Diaphragm, pectoralis, and abdominal muscles	Limb muscles
Microarray	MOE430A (Affymetrix)	MOE430v2 (Affymetrix)	MOE430v2 (Affymetrix)
Quiescence genes^*^			
Calcr	QSC>ASC (×74)	QSC>ASC (×1.8)	ND
Nap1l5	QSC>ASC (×62)	QSC>ASC (9.9)	ND
Odz4	QSC>ASC (×24)	QSC>ASC (×1.96)	ND
Pde4b	QSC>ASC (×23)	QSC>ASC (×5.77)	QSC>ASC (×11.21)
Pof1b	QSC>ASC (×23)	QSC>ASC (×2.79)	ND
RhoH	QSC>ASC (×17)	QSC>ASC (×3.9)	ND
Cebpb	QSC>ASC (×16)	QSC>ASC (×3.19)	ND
Maff	QSC>ASC (×14)	QSC>ASC (×3.19)	ND
Quiescence genes^**^			
Apoe	QSC>ASC (×293)	QSC>ASC (×23)	ND
Dpt	QSC>ASC (×199)	QSC>ASC (×2.2)	ND
Ms4a4d	QSC>ASC (×144)	QSC>ASC (×78)	ND
Adh1	QSC>ASC (×55)	QSC>ASC (×28)	ND
Osmr	QSC>ASC (×37)	QSC>ASC (×26)	ND
Fgl2	QSC>ASC (×23)	QSC>ASC (×42)	ND
Timp4	QSC>ASC (×12)	QSC>ASC (×49)	ND
Ahr	QSC>ASC (×8)	QSC>ASC (×48)	ND
**Cell adhesion**	Chodl, Eva1, CD34, Aoc3, Tek, Icam1, Pcdhb9. Efs, Esam1, Cdh5, Cldn5, Nope, Itgb5, Emcn, Cdh13, Sdc4, Dcn, Pcdh9n	Itgb1, CD24, Sdc4, Cldn1, Eva1, CD38, Esam1, Smoc2, Icam2, Cldn5, Dcn, Fgl2	Sdc4, Pak1, Pcdh18, Col3a1
**Notch**	QSC high (Notch3, HeyL)	QSC low	QSC high (HeyL)
**Anti-oxidative stress**	Cyp4b1, Gstt1, Gstt3, Sod3, Ugt1a2, Ahr, Cp, Gpx3, Cyp2d22, Cyp39a1, Cyp27a1	Sult1a1, Cyp4b1, Abcd1a, Ugt1, Fmo2, Ahr, Gstm1, Cyp26b1, Srxn1, Txnrd1, Cp, Gpx3	ND
**Transcriptional factors**	Sox17, Meox2, Sox7, Sox18, Klf4, 9, 15	Sox17, Meox2, Sox7, Sox18, Klf7, Dach1, Prdm16	ND
**RNA-binding protein**	Rnase4, Zfp36, Zfp36l1, Rnpc2, Csdc2	Rnase4, Ddx5, Sfrs3, Son, Zfp36, Ddx58, Rbpms	Cherp, Ddx5, Hnrnpc, Sfrs3, Snrpb, Snrpe, Son, Srrm1, U2af1l4, Zranb2, Eif4h, Hnrpdl, Poldip3, Rpl22, Rps23, Rps9, Khdrbs1, Rnaset2a, Zfp36, Zfp36l1, Ddx58, Mex3b, Qars, Rad21, Rbm26, Rbpms

ND, Not described.

* “Quiescence genes” of our analyses. These genes are highly expressed in quiescent satellite cells more than in non-myogenic cells in skeletal muscle.

** These genes are highly expressed in quiescent satellite cells, but not listed in our “quiescence genes” because non-myogenic cells also expressed these genes.

### Notch effector genes and satellite cells

Notch signaling is essential for development of diverse tissues (Lai, 2004). When Notch is activated, its intracellular domain is cleaved by γ-secretase and it translocates to the nucleus, where it activates the transcription of target genes through interaction with Rbpj. Rbpj-mediated Notch signaling is known as the canonical pathway, and the families of *Hes* (hairy and enhancer of split) and *Hesr* (hes-related, also known as Hey/Herp/Hrt/Chf) are known as primary targets of Notch signaling (Iso et al., 2003; Fischer and Gessler, 2007). Among Hes and Hesr family genes, quiescent satellite cells specifically and highly express Hesr3 in skeletal muscle. Quiescent satellite cells also express Hesr1. However, Hesr1 is not included in the ‘quiescence genes’ because endothelial cells in skeletal muscle, as well as other tissues, express Hesr1. In addition, when primary myoblasts or C2C12 are stimulated with Delta-like 1 or 4, respectively, Hesr1 and Hesr3 are induced in both types of cells (Buas et al., 2009; Fukada et al., 2011). Therefore, Hesr1 and Hesr3 seem to be the major downstream targets of Notch signaling in adult satellite cells (Yamaguchi and Fukada, 2013).

The roles of Notch signaling are powerful and complicated because it has opposite effects on some lineage cells. In myogenic cells, Notch signaling has two roles: one is inducing myogenic commitment and the other is inhibiting myogenesis. Dezawa et al. produced myogenic cells from bone marrow stromal cells via the transient activation of Notch signaling in the processes (Dezawa et al., 2005). Rios et al. reported that neural crest-derived delta-like 1 transiently activates Notch signaling in cells located in the medial border of the dermomyotome and that this event is essential for the induction of both Myf5 and MyoD in them (Rios et al., 2011). They also showed that sustained Notch signaling inhibits myogenic differentiation even in the medial border of the dermomyotome. These results demonstrated that transient Notch signaling works as an inducer of myogenesis. On the other hand, the myogenic inhibitory effect of Notch signaling is extremely well known (Kuroda et al., 1999). Induction of Notch signaling suppresses the expression of MyoD, and myogenic differentiation is strongly inhibited. Hesr1 and Hesr3 also seem to play roles in anti-myogenic differentiation because unusual expressions of MyoD and myogenin were observed in satellite cells derived from Hesr1/Hesr3 double-knockout mice (Fukada et al., 2011). Although Hesr1 or Hesr3 single-knockout mice did not show any defect in skeletal muscle including satellite cells and regenerative potential, Hesr1/Hesr3 double-knockout mice showed a remarkable defect in satellite cells. Therefore, in adult satellite cells, Notch signaling seems to be activated constitutively to work as a myogenic inhibitor.

Vasyutina et al. demonstrated the essential roles of canonical Notch signaling to generate the satellite cell pool during embryonic development using conditional depletion of Rbp-J (Vasyutina et al., 2007). However, in Hesr1/Hesr3 double-knockout mice, most of the satellite cell pool existed in mice by the 7th day after birth. These results suggest that the downstream target of Notch is changed during skeletal muscle development. Using Rbpj-floxed and Pax7-CreERT2 mice, two independent groups reported the essential role of Notch signaling for maintaining satellite cells in an undifferentiated state in mouse adult skeletal muscle (Bjornson et al., 2012; Mourikis et al., 2012). When Rbpj was depleted in satellite cells by injection of tamoxifen, quiescent satellite cells started to express myogenic proteins (MyoD and myogenin) and then fused with myofibers. Although further study of conditional Hesr1/Hesr3 depletion remains to be done, these results suggest that the Notch/Rbp-J/Hesr1/Hesr3 pathway is essential to maintain adult satellite cells in a quiescent and undifferentiated state (Yamaguchi and Fukada, 2013). In Table 2, we summarized the phenotypes of Rbp-J-null and Hesr1/Hesr3-dKO mice in adult skeletal muscle.

Melanocyte, intestinal, and neural stem cells also use canonical Notch signaling for their maintenance (Moriyama et al., 2006; Imayoshi et al., 2010; Pellegrinet et al., 2011). In these three stem cells, Hes1 is the major downstream target of Notch signaling. Although the downstream target of Notch signaling is not conserved, canonical Notch signaling seems to be a common molecular mechanism to maintain stem cells in some adult tissues. Notch signaling is essential for the differentiation of hematopoietic cells, but hematopoietic stem cells do not require Notch signaling for maintenance (Maillard et al., 2008).

As described, Notch signaling seems to be one of the essential signaling pathways for maintaining satellite cells. The activation of Notch signaling is induced by its specific ligands, Dll1, Dll4, and Jagged. Basically, direct cell–cell contact is necessary to induce Notch signaling. Until now, the ligand and its origin for maintaining the satellite cell pool have been unclear. Satellite cells are directly attached to myofibers, and therefore, myofibers may express the ligand. Another possibility is that released ligand activates Notch signaling in satellite cells. Sun et al. indicated that Dll1 can be shed and act on myoblasts in an autocrine manner (Sun et al., 2008). The other possibility is ligand-independent activation of Notch receptors. Sima protein, an ortholog of mammalian hypoxia-inducible factor-α (HIF-α), colocalizes with Notch in endocytic vesicles and enables cleavage of the intracellular domain of Notch in Drosophila blood cells (Mukherjee et al., 2011). Gustafsson et al. also demonstrated that HIF-α interacts with the Notch intracellular domain and promotes the expression of the Notch target genes (Gustafsson et al., 2005). The role of HIF-α in muscle satellite cells is still unknown, but these pathways may be used to activate Notch signaling in adult satellite cells.

### Notch signaling and other signaling pathways

Cross-talk signals between Notch and other pathways in some types of cells are reported. For example, Hes proteins promote Stat3 phosphorylation and activation through association with Jak2 and Stat3 in neuroepithelial cells (Kamakura et al., 2004). BMP and Notch signaling also have synergistic effects. Dahlqvist demonstrated that BMP-induced inhibition of myogenic differentiation requires Notch signaling (Dahlqvist et al., 2003). However, phosphorylation of neither Stat3 nor Smad1/5/8 (downstream targets of BMP signaling) was observed in quiescent satellite cells (Kami and Senba, 2002; Wang et al., 2010). Therefore, these signaling pathways do not seem to work coordinately with Notch signaling to sustain the satellite cell pool, although they might work together to activate or start proliferation of satellite cells because phosphorylated Stat3 nor Smad1/5/8 are observed in activated or proliferating satellite cells.

In endothelial cells, Notch signaling inhibits the phosphorylation of Rb (a driver of cell cycle progression), which leads to a decrease in BrdU uptake (Noseda et al., 2004). Rb is a well-known tumor suppressor gene, and phosphorylation of Rb allows the cell cycle to progress. Hosoyama et al. demonstrated that Rb-conditional depletion increased the number of satellite cells in uninjured muscle (Hosoyama et al., 2011). Therefore, even in quiescent satellite cells, Notch signaling might inhibit the phosphorylation of Rb to suppress cell cycle progression. However, the expression of proliferative markers (Ki67 and phosphorylated histone-H3) was not observed in Rb-cKO satellite cells, as it was in Rbp-J cKO satellite cells. Therefore, these results suggest that Rb-independent quiescence mechanisms are regulated by Notch signaling, which plays roles in maintaining the satellite cell pool in dormant state.

Ge et al. reported that Smad3-null mice had a decreased number of satellite cells (Ge et al., 2011). Using the myogenic cell line C2C12, Blokzijl et al. indicated that TGF-β stimulation induces Hes1 expression in a Notch signaling activation-dependent manner (Blokzijl et al., 2003). Furthermore, Smad3 interacts directly with NICD and binds to the promoter regions of Notch target genes via Rbp-J. Therefore, in quiescent satellite cells, TGF-β and Notch might work cooperatively to sustain the dormant state.

### Calcitonin receptor and Odz4 and satellite cells

Calcitonin is a molecule that is well known to regulate homeostasis of the calcium level in the blood (Becker et al., 2002). Calcitonin is released from the thyroid and works in bone and kidney. The action of calcitonin is mediated by its specific receptor, the calcitonin receptor. The calcitonin receptor is a G-protein-coupled seven transmembrane protein. Expression of calcitonin receptor is well known in osteoclasts, which play essential roles in bone absorption, and results in the release of calcium into the blood. The balance between osteoclasts and osteoblasts is tightly regulated to maintain bone homeostasis. When the balance is tipped toward osteoclasts, the osteoclastic process is accelerated, and osteoporosis occurs. Calcitonin receptor signaling inhibits the function of osteoclasts via protein kinase A (Suzuki et al., 1996); therefore, synthetic calcitonin is used for treatment of osteoporosis. We found specific expression of calcitonin receptors in quiescent satellite cells, but not in activated satellite cells. The expression of calcitonin receptors seems to be specific in quiescent satellite cells because the re-expression of calcitonin receptors during the regenerative process is related to the end of muscle regeneration (Fukada et al., 2007; Yamaguchi et al., 2012). Therefore, these specific expression patterns imply that calcitonin receptors play several roles in maintaining satellite cells in a quiescent state. In fact, we observed that activation of calcitonin receptors by its ligand delays activation of satellite cells *in vitro* (Fukada et al., 2007). Calcitonin receptor-null mice die *in utero*, and thus the roles of calcitonin receptors in myogenic lineage cells remain unknown. To reveal the physiological importance of calcitonin receptors in satellite cells, the study of satellite cell-specific deletion of calcitonin receptors is essential, and is one of our most important ongoing investigations.

Although the physiological importance of calcitonin receptors in satellite cells is unclear, Cheung et al. demonstrated that mir-489, which is located in intron 4 of the *calcitonin receptor* gene, is essential for satellite cell quiescence (Cheung et al., 2012). Mir-489 is also specifically expressed in quiescent satellite cells like calcitonin receptor mRNA. Because coding and non-coding genes are often transcribed simultaneously, transcription of *calcitonin receptor* genes is tightly regulated in satellite cells, and investigations of *calcitonin receptor* gene regulation might elucidate the activation or self-renewal mechanism of satellite cells.

**Figure 2:**
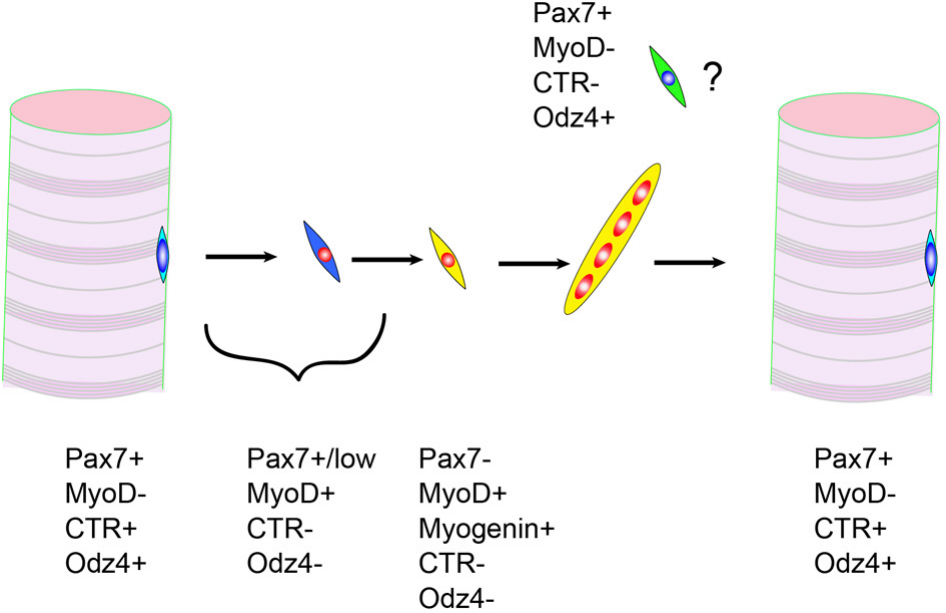
**Expressions of calcitonin receptor (CTR) and Odz4 proteins during skeletal muscle regeneration**. Pax7+MyoD-CTR-Odz4+ cells are frequently observed during myofiber maturation.

Odz is the vertebrate homolog of the Drosophila odd Oz. Odz family proteins belonging to the type II transmembrane protein family (Levine et al., 1994). One member of the Odz family, Odz4, is highly expressed in the central nervous system, developing eyes, and somites (Zhou et al., 2003). In addition, we reported the expression of Odz4 protein in satellite cells (Yamaguchi et al., 2012). The function of Odz4 is little known, but recent reports demonstrated the importance of Odz in oligodendrocyte differentiation and process formation (Suzuki et al., 2012). They also indicated that focal adhesion kinase, a key regulator of cell adhesion, is activated downstream of Odz4. Therefore, in satellite cells, Odz4 might control cell adhesion and/or differentiation. Odz4 and calcitonin receptor are expressed in quiescent satellite cells but not in proliferating myoblasts. Intriguingly, the timings of Odz4 and calcitonin receptor re-expression during skeletal muscle regeneration are different (Yamaguchi et al., 2012). Currently, we do not know the characteristics of Pax7(+)Odz4(+)calcitonin receptor(−) cells, but we have speculated that this type of cell contributes to the maturation of myofibers because the appearance of Pax7(+)Odz4(+)calcitonin receptor(−) cells is observed during maturation of immature myofibers (Figure 2). Like *calcitonin receptor* genes, the *Odz4* intron contains a quiescent satellite cell-specific microRNA, mir-708 (Cheung et al., 2012). The function of mir-708 is also unknown, but the gene expression mechanism of *Odz4* might be important for efficient skeletal muscle regeneration.

### Bone and skeletal muscle may use common molecules for maintenance

Skeletal muscle works as a locomotorium in cooperation with bone. Some diseases or environmental conditions affect both skeletal muscle and bone. For instance, inactivity, as seen in a gravity-free state or bed rest, leads to the loss of muscle weight and bone density. Aging and muscular dystrophy also affect both skeletal muscle and bone states. Duchenne muscular dystrophy patients have low bone mineral density and increased risk of fractures (Bachrach, 2005). Although it has been considered that the bone impairment of DMD patients results from muscle weakness, the bone-skeletal muscle system might have direct regulatory networks via cytokines or exosomes. Otherwise, the bone and skeletal muscle systems use a similar gene network to maintain their homeostasis.

Hesr1/Hesr3 are essential for generation of quiescent satellite cells and to maintain their numbers in adult skeletal muscle. Hesr1/Hesr3 double knock-out mice have other phenotypes besides that of satellite cells. Fischer et al. reported that deletion of both Hesr1 and Hesr3 causes severe heart malformations, including membranous ventricular septal defects and dysplastic atrioventricular and pulmonary valves (Fischer et al., 2007). In addition, Tu et al. demonstrated the essential roles of Hesr1/Hesr3 in osteoblasts (Tu et al., 2012). As is well known, expression of calcitonin receptors in osteoclasts is essential to sustain the homeostasis of bone. Although the physiological importance of calcitonin receptors remains unknown, Hesr1/Hesr3 and calcitonin receptor might be common regulators for both bone and skeletal muscle systems.

### Quiescence genes and cancer progression

The quiescent state includes two types of cell cycles, reversible and irreversible. Myofibers are mitotically quiescent, and it is an irreversible state. The induction of msx1 in myotubes (which are also irreversibly quiescent) leads to the generation of mononuclear cells that proliferate (Odelberg et al., 2000). This phenomenon is biologically interesting, but myotubes and myofibers do not generate mononuclear cells in a physiological manner. On the other hand, stem cells, including satellite cells, are in a reversible quiescent state. The molecular mechanisms of reversible quiescence are an interest of many investigators. Reversible quiescence is conserved from yeast to human cells to maintain particular cells, including stem cells, in the body. Uncontrolled reversible quiescence mechanisms can lead to cancer. Intriguingly, Spry1, Notch3, Cebpb, calcitonin receptor, and Odz4 are reported to relate to cancer cells (Wang et al., 1999; Kwabi-Addo et al., 2004; Thomas and Shah, 2005; Park et al., 2006). Cell cycle inhibitors (cyclin-dependent kinase inhibitors) are highly expressed in stem cells, and their breakdown leads to abnormal cell cycle regulation and results in cancer development. Sang et al. indicated that Hes1 is a key transcriptional factor for reversible cellular quiescence in human fibroblasts (Sang et al., 2008). The authors also showed that Hes1 allows human rhabdomyosarcoma cells to evade differentiation and irreversible cycle arrest. As described above, melanocyte stem cells, intestinal stem cells, and neural stem cells require Hes1 downstream of Notch signaling to maintain their pool. Therefore, elucidation of the quiescence mechanisms of stem cells, including satellite cells, will shed light on the principle of the quiescent state, and may lead to the discovery of new therapeutic targets for cancers.

## Relationship between satellite cells and muscle disorders

Muscular dystrophies are the best known muscle disorders, and investigations of them have been central to exploring skeletal muscle biology. The loss of satellite cell numbers and function is considered to be one reason why many muscular dystrophies exhibit progressive symptoms. Besides muscular dystrophies, there are many muscle disorders that exhibit a relationship with impairments of satellite cells. In addition, some reports have shown the direct contribution of satellite cells to the disease condition. For instance, the appearance of fibrosis and adipocytes had been considered to be due to satellite cell transdifferentiation into fibroblasts and adipocytes (Asakura et al., 2001; Li et al., 2004; Alexakis et al., 2007). However, recent studies have demonstrated that satellite cells cannot differentiate into adipocytes and fibroblasts (Joe et al., 2010; Uezumi et al., 2010, 2011; Starkey et al., 2011). Consistent with these observations, Crist et al. clarified that satellite cells are committed progenitor cells especially linked to myogenic cells (Crist et al., 2012). In this section, we will focus on the relationship between satellite cell myogenic potential and disease progression in three muscle disorders and discuss the contribution of satellite cell function to these disorders.

### Difference between human muscular dystrophy and mouse model

Duchenne muscular dystrophy (DMD) is a well-known inherited muscular disorder, and the causative gene, *Dystrophin*, is coded in the X-chromosome (Koenig et al., 1988). Patients exhibit progressive symptoms, and histologically, accumulation of fibrosis and adipocytes and loss of myofibers are observed. The *Dystrophin* gene encodes a 427-kDa cytoskeletal protein that forms the dystrophin/glycoprotein complex at the sarcolemma with α- and β-dystroglycans, α-, β-, γ-, ε-, and δ-sarcoglycans, and other molecules, and links the cytoskeleton proteins of myofibers to the extracellular matrix in skeletal muscle (Ervasti and Campbell, 1993). It is supposed that the loss of dystrophin leads to the degeneration of myofibers due to a disturbance in assembly of the dystrophin/glycoprotein complex.

The *mdx* mouse (the correct nomenclature is C57BL/10-DMDmdx) is the most widely used model animal of DMD (Bulfield et al., 1984). Although *mdx* mice have a mutation in the *dystrophin* gene and show degeneration of myofibers, the symptoms of *mdx* mice are remarkably milder than those of DMD patients. In contrast with DMD patients, accumulation of fat and fibrosis in *mdx* mice are barely observed except in the diaphragm, and neither myofibers nor muscle weight are lost throughout much of their life span. One reason for the difference between DMD and *mdx* is explained by the excellent regeneration capacity of *mdx* compared with DMD. However, *mdx* mice carrying another strain background (DBA/2) show similar phenotypes to humans; loss of muscle weight, increased fibrosis, accumulation of adipocytes, and decreased muscle force (Fukada et al., 2010). Intriguingly, satellite cell functions of DBA/2 also differ from those of C57BL/6 mice. Therefore, one of reason for the severe phenotype of DBA/2-*mdx* seems to be inferior function of satellite cells compared to C57BL/10-*mdx*.

The life spans of mice and humans are completely different. In humans, long-term proliferation of satellite cells might be necessary. However, the human telomere is much shorter than that of the mouse. Based on this, Sacco et al. hypothesized that the longer telomere allows satellite cells to proliferate repeatedly in mice. To elucidate this hypothesis, they generated *mdx* mice lacking the RNA component of telomerase (*mdx*/mTR) and demonstrated that *mdx*/mTR mice exhibit severe muscular dystrophy and a decrease in the loss of satellite cell proliferation (Sacco et al., 2010). The phenotypes of DBA/2-*mdx* are unlikely dependent on the telomere erosion because DBA/2 mice have longer telomeres than C57BL/6 (Manning et al., 2002). These results indicate that the differences between human and mouse models depend on several functions of satellite cells: one is telomere length, but the other factors are unknown.

Other dystrophic mouse models, gamma-sarcoglycan-null mice, also depend on the mouse genetic background, and DBA/2-background mice exhibit the most severe phenotype of the strains examined (Heydemann et al., 2005). Intriguingly, the aged phenotype of DBA/2 is much more severe than that of C57BL/6 (Lionikas et al., 2006). In addition, the low reconstitution potential of the DBA/2-strain is not restricted to skeletal muscle. DBA/2-derived hematopoietic stem cells (HSCs) show low reconstitution, and Liang et al. revealed the gene, latexin, responsible for this phenotype (Liang et al., 2007). These results suggest that similar analyses will also lead to the discovery of genes responsible for skeletal muscle satellite cells, which may lead to the discovery of a new therapeutic methodology for muscular disorders.

### Muscular dystrophy and satellite cells

Conceptually, exhaustion of the satellite cell pool leads to the progression of muscular dystrophy. In fact, the loss of proliferative potential was observed in myoblasts derived from DMD patients (Blau et al., 1983b). One of our surprising findings was that quiescent satellite cells showed high expressions of *dystrophin* and *dystroglycan* genes compared to myoblasts (Fukada et al., 2007). Both dystrophin and dystroglycan have been considered essential proteins for stability of the myofiber membrane (sarcolemma), and it is well known that the lack of these genes leads to muscular dystrophies. The roles of these genes in satellite cells are still unknown, but these genes might be essential for cell adhesion and stability of satellite cells as well as myofibers.

Recently, Kanagawa et al. showed a functional defect of myogenic cells in the mouse model of Fukuyama-type congenital muscular dystrophy, which is caused by an ancient retrotransposal insertion in the *Fukutin* gene (Kobayashi et al., 1998; Kanagawa et al., 2013). Interestingly, fukutin-deficient myoblasts showed a significantly low potential for myotube formation, even myoblasts that originated from mice that had not started to exhibit a dystrophic phenotype. These results indicate that fukutin is necessary for myotube formation; therefore, efficient regeneration is likely to be impaired in Fukuyama-type muscular dystrophy. In addition, lamin A/C and emerin, which are expressed in quiescent satellite cells, are known as causal genes for autosomal-Emery-Dreifuss muscular dystrophy (A-EDMD) (Bonne et al., 1999) and X-EDMD (Bione et al., 1994), respectively. Therefore, some causative genes for muscular dystrophy may affect satellite cells and/or their daughter cells directly, which might determine the severity of symptoms (Gnocchi et al., 2008).

### Sarcopenia and satellite cells

Current progress and improvement of medical treatment prolong lives, but aging-related physical morbidity is becoming a social problem. Normal skeletal muscle also faces these problems. Most of us exhibit drastic deterioration of performance with age. One cause is sarcopenia, which is linked to the loss of muscle mass and function. Sarcopenia is inevitable, and likely to contribute to the decline of muscle strength and ability to maintain daily activities. Some researchers define sarcopenia as “an appendicular muscle mass/height^2^ less than two standard deviations below the mean for that of a young healthy adult” (Iannuzzi-Sucich et al., 2002). According to this definition, the percentage of elderly suffering from sarcopenia has reached 10–25% or more (Baumgartner et al., 1999). In light of this severe phenomenon and the tendency of the global geriatric population to increase in number, sarcopenia will become a social problem. Besides sarcopenia, there are other muscle disorders accompanying the loss of muscle mass, known as atrophies. However, sarcopenia can be distinguished from other types of muscle atrophy by a decrease in the number of myofibers. Myofiber formation is based on the fusion of a large number of myoblasts (Moss and Leblond, 1970; Hawke and Garry, 2001) and requires the participation of satellite cells in the regenerative process of myofibers; a relationship between satellite cells and sarcopenia is possible, although it still remains controversial. Trendelenburg et al. demonstrated that TAK-1/p38/nNF-κB signaling pathway inhibits myoblast differentiation by increasing the level of activin A (Trendelenburg et al., 2012). Upregulation of TNF-1α and IL-1β is reported in sarcopenia, and they drive TAK-1/p38/nNF-κB. The NF-κB pathway is well known as an inducer of muscle atrophy (Cai et al., 2004). Therefore, the NF-κB pathway might be activated in both satellite cells and myofibers, which leads to suppression of myogenic differentiation and atrophy in myofibers.

In sarcopenia, direct contributions by the satellite cell pool are proposed because many studies have demonstrated the loss of satellite cell pools throughout the aging period. In contrast, some reports indicate that the number of satellite cells in muscle of elderly rats and mice is unchanged. Although it has not been fully elucidated, the discrepancy is likely to be connected to a difference in the muscle tissues analyzed (e.g., levator, soleus, vastus lateralis, and tibialis anterior, etc.) (Gibson and Schultz, 1983; Nnodim, 2000; Brack et al., 2005; Schafer et al., 2005). Based on the fact that sarcopenia mainly presents a decline in type 2 muscle fibers, some researchers have confirmed the observation of a 45% reduction in satellite cell numbers in type 2 muscle fibers in old (76 ± 1 years) vs. young (20 ± 1 years) populations (Verdijk et al., 2007). In addition, several available tests demonstrate that activated satellite cells can partially counter sarcopenia (Cutlip et al., 2009; Snijders et al., 2009; Aagaard et al., 2010). These backgrounds contribute to the maximal connection between satellite cells and sarcopenia. Intriguingly, DBA/2 strain mice exhibit a more remarkable loss of muscle mass than C57BL/6 mice, and the satellite cell pool of DBA/2 is reduced earlier in their life span than that of C57BL/6 (our unpublished data). As described in the previous section, DBA/2 satellite cells are inferior to those of C57BL/6 mice, and therefore, the investigation of DBA/2-satellite cells might lead to discovery of the central pathway for the maintenance of satellite cell pool throughout life.

### Cancer cachexia and satellite cells

Some cancers induce the loss of body weight. A decrease beyond 5% of body weight in 12 months or less can be defined as cancer cachexia. Because skeletal muscle mass occupies about 40% of body weight, cancer cachexia is generally associated with the loss of muscle weight. Although the molecular mechanism evoking muscle atrophy in cancer cachexia patients remains largely unknown, some studies have begun to reveal the molecular mechanisms of cancer cachexia. Acharyya et al. indicated that dysfunction of dystrophin in a cancer cachexia mouse model leads to weakness of the myofiber membrane (Acharyya et al., 2005). The membrane structure of myofibers is considered an important element for maintaining the satellite cell pool, and therefore, there is a possibility that a change in the myofiber membrane affects satellite cells. In fact, Penna et al. found an increased number of satellite cells in cancer cachexia model mice (Penna et al., 2010). Further, up-regulation of TNF-α and IL-6 in cachexia patients and animal models is well known. Although the relationship between these cytokines and muscle wasting is unclear, these cytokines inhibit muscle differentiation (Coletti et al., 2002, 2005; Guttridge, 2004). Zhou indicated that activation of ActRIIB (receptor for activin and myostatin) contributes to the loss of mass in cancer cachexia model mice and that an ActRIIB antagonist may be a therapeutic approach to the treatment of cancer cachexia (Zhou et al., 2010). As described for sarcopenia, the ActRII signaling pathway inhibits myogenic differentiation. Based on this information, it is possible to hypothesize that cancer cachexia involves satellite cell dysfunctions. In this case, satellite cells may be directly targeted for the treatment of cancer cachexia, and a molecular mechanism for regulation of satellite cells may solve this problem in the near future. In addition, recently it was reported that muscle stromal cells (PDGFRa+ cells, which are considered to be mesenchymal progenitors) are essential to sustain muscle mass (Roberts et al., 2013). Depletion of muscle stromal cells leads to the loss of muscle mass, and cancer cachexia induced a decrease in the number of muscle stromal cells. Therefore, muscle mesenchymal progenitors might be also a direct target of cancer cachexia as well as muscle satellite cells and myofibers.

## Future strategies for stem cell research to treat muscle disorders

Satellite cells undoubtedly have the best potential to produce new myofibers *in vivo*. However, there are obstacles to overcome. One problem is the low migration potential of satellite cells. Most muscular dystrophy patients have dystrophic symptoms in systemic skeletal muscles; therefore, satellite cells have to be transplanted via blood vessels. Unfortunately, satellite cells cannot cross blood vessels, so the use of satellite cells is considered limited to the relatively localized muscle diseases such as oculopharyngeal muscular dystrophy (OPMD). Recently, Cappellari et al. reported that Dll4 and PDGFβ signals convert myogenic cells to pericyte-like cells without erasing their myogenic memory (Cappellari et al., 2013). Unlike satellite cells, pericytes can cross blood vessel walls and migrate into skeletal muscle tissue (Dellavalle et al., 2007). Therefore, satellite cells might acquire the ability to cross blood vessels and form myofibers by using the methodology.

Another problem is the difficulty of obtaining large numbers of satellite cells from a donor. In addition, expansion of satellite cells *in vitro* reduces their regenerative activity, as described above (Montarras et al., 2005; Ikemoto et al., 2007). However, several studies have shed light on ways to improve the culture system. Gilbert et al. indicated the importance of substrate elasticity in sustaining satellite cell function *in vitro* (Gilbert et al., 2010). Notch ligand stimulation also allows the expansion of satellite cells without a decrease *in vivo* regenerative potential (Parker et al., 2012). As described above, recent studies have indicated that Notch signaling is one of the essential signaling pathways to maintain satellite cells. The reason why Notch signals improve myogenic cell transplantation is unknown, but one possibility is the down-regulation of MyoD. Asakura et al. demonstrated that the survival of MyoD-null mice-derived myoblasts is superior to that of wild-type myoblasts (Asakura et al., 2007). The authors also showed that many anti-apoptotic genes were up-regulated in MyoD-null myoblasts, whereas genes known to execute apoptosis were down-regulated. The relationship between Notch and MyoD in an improved protocol for myogenic cell transplantation must be revealed for realization of satellite cell therapy for DMD patients because Notch signaling also affects cell cycle genes in satellite cells.

Another source of cells for therapy for muscle disorders is iPS cell-derived myogenic cells (Mizuno et al., 2010; Darabi et al., 2012). A method to produce myogenic cells from iPS cells, which have some of the same features as satellite cells, might solve the problem of cell numbers. Because satellite cells are mitotically quiescent, we need to generate proliferating myogenic cells that have myogenic potential similar to satellite cells. The myogenic cell appearing in embryonic and neonatal developmental stages is one model candidate for a myogenic progenitor derived from iPS cells. However, Sakai et al. compared the regenerative potential of fetal and adult satellite cells, and found that adult satellite cells are superior to fetal satellite cells (Sakai et al., 2013). Researchers have just begun to understand the satellite cell on the molecular level. For the development of satellite cell-like cells from iPS cells, we need to understand the process of generating satellite cells during development as well as regeneration. Relaix et al. observed that Pax3(+)Pax7(+) cells derived from the central region of the dermomyotome are the origin of satellite cells (Relaix et al., 2005). In addition, satellite cells express MyoD during this process (Kanisicak et al., 2009). The Notch signaling pathway perhaps down-regulates MyoD to generate the satellite cell pool. However, before they become myogenin(+) cells, we cannot anticipate the destiny of MyoD(+) myogenic cells during the development and regeneration processes. One of the most important questions is how the satellite cell pool is established during embryonic and postnatal development. Understanding this will lead to success in the generation of satellite cell-like cells from iPS cells.

The physiological self-renewal mechanism of satellite cells during regeneration must also be revealed. Some evidences have indicated an asymmetrical model of self-renewal of satellite cells (Conboy and Rando, 2002; Shinin et al., 2006; Kuang et al., 2007). Wnt7a signaling promotes symmetrical division of Myf5- satellite cells and ameliorates the *mdx* phenotype (Le Grand et al., 2009; von Maltzahn et al., 2012). Elucidation of these processes may also lead to successful generation of satellite cell-like cells from iPS cells. Taken together, in either case (satellite cells or iPS cells), the molecular mechanisms for both generation, maintenance, and self-renewal of satellite cells must be revealed to attain our goal.

In addition, another mechanism must be understood for the successful cell transplantation for muscle disorders. To date, we have not paid attention to the fusion process between transplanted cells and myofibers. When donor cells are transplanted, they have to cross the basal lamina and fuse with myofibers. Horsley et al. indicated the myotube-myoblast fusion process requires IL4/IL-4R signaling mediated by NFATc2 (Horsley et al., 2003). However, the structure, functions, and gene expressions of myotubes differ remarkably from those of myofibers. Therefore, we need to consider the process of myoblast-with-myofiber fusion. The identification of such genes will improve the efficiency of satellite cell/iPS-derived myogenic cell therapy.

## Prospects

For 10 years, the isolation, characterization, and molecular regulation of satellite cells have been studied, and accumulating evidence is starting to reveal the molecular mechanisms for maintaining the satellite cell pool. In addition, recent studies are beginning to identify the roles of satellite cells in several different disorders. For instance, McCarthy et al. indicated that acute hypertrophy is not dependent on satellite cells (McCarthy et al., 2011). On the other hand, acute and chronic regenerations are required to fulfill the potential of satellite cells. These results indicate that not all muscle-related disorders depend on satellite cell function. In the aging process, the dependence on satellite cells is controversial. To develop a new therapeutic approach, we have to understand the contribution of satellite cells to each disease. In sarcopenia research, a Pax7-CreERT2::Rosa-DTA strategy might be useful to elucidate the dependency of satellite cells. In addition, until now, the genes causing muscular dystrophies have only been considered as a function of the sarcolemma in myofibers. However, some of them are expressed in quiescent satellite cells (Cohn et al., 2002; Fukada et al., 2007; Gnocchi et al., 2009). Therefore, they may regulate the quiescence and undifferentiated states of satellite cells, and additional functions of causative genes in satellite cells might explain the severity of each disease.

The generation of iPS cells has assisted the cell therapy approach to many disorders including muscular dystrophy (Takahashi and Yamanaka, 2006; Takahashi et al., 2007). The successful establishment of iPS cells was supported by studies of ES cells. Finding culture conditions that can maintain ES cells in an undifferentiated state is probably the most important contribution to the birth of iPS cells. For cell treatment of muscular disorders, we have at least two choices, satellite cells or iPS-derived myogenic cells. In either case, we would like to reiterate that understanding the molecular mechanisms of muscle satellite cells is essential to accomplish successful myogenic cell therapy.

Skeletal muscle is indispensable for motility. In addition, skeletal muscle has the potential to control other tissues. For instance, Bostrom et al. showed that a skeletal muscle-derived cytokine, irisin, converts white fat cells to brown fat-like cells (Bostrom et al., 2012). The relationship between exercise and immune function is a well-known open window theory (Nieman and Pedersen, 1999). Thus, skeletal muscle itself might be a therapeutic target for other diseases like diabetes. Much still remains to be revealed about striated muscle, but striated muscle might have many powers to control unanticipated physiologies. We hope that the study of satellite cells will open new doors of striated muscle biology and lead to a recovery of muscle power in muscle disorder patients.

### Conflict of interest statement

The authors declare that the research was conducted in the absence of any commercial or financial relationships that could be construed as a potential conflict of interest.
